# Disparities in Perceived Availability of Healthful Foods, Dietary Behaviors, Diet Quality, and Obesity Among Mothers from Low-Income Households: Additional Evidence in the Call for Broader Approaches to Obesity Prevention

**DOI:** 10.1089/heq.2022.0127

**Published:** 2023-04-20

**Authors:** Fred Molitor, Sarah Kehl

**Affiliations:** ^1^ Department of Communication Studies, California State University Sacramento, Sacramento, California, USA.; ^2^ CalFresh Healthy Living Program, California Department of Social Services, Sacramento, California, USA.

**Keywords:** mothers, SNAP-Ed, low-income, disparities, nutrition, obesity, prevention, diet quality

## Abstract

**Purpose:**

To examine racial/ethnic differences in dietary behaviors, diet quality, body mass, and the perceived availability of healthful foods in one's neighborhood among mothers from low-income California households.

**Methods:**

Cross-sectional telephone surveys of mothers from randomly sampled households with incomes ≤185% federal poverty level in 2018 and 2019 using a validated 24-h dietary recall assessment. Dietary outcomes were cups of fruits and vegetables, ounces of sugar-sweetened beverages, teaspoons of added sugars, and kilocalories consumed the previous day. Diet quality was assessed by calculating Health Eating Index-2015 scores. Supplemental survey items assessed mothers' weight and height. Body mass index (BMI) was calculated with a BMI of 30 or higher considered obese. Perceived availability of fresh fruits and vegetables and healthy foods in general within one's neighborhood was recorded.

**Results:**

The analytic sample of 9200 mothers was 66.3% Latina, 17.3% white, 12.6% African American, and 3.8% Asian American, Native Hawaiian, or Pacific Islander (AANHPI). African American mothers consumed the fewest cups of fruits and vegetables and the most teaspoons of added sugars, reported poor diet quality, and had the highest obesity rate, 54.7% versus 46.9% for Latinas, 39.9% for whites, and 23.5% for AANHPIs. Accordingly, a greater proportion of African Americans reported limited availability of fresh fruits and vegetables and healthy foods in general in their neighborhood.

**Conclusion:**

Findings are interpreted in light of recent calls for broader approaches to address health disparities, including strategies that focus on inequalities in racial/ethnic socioeconomic status and systemic racism.

## Introduction

The risk of negative health and quality of life outcomes are elevated for individuals with excessive body weight. The consequences of overweight and obesity include an increase in the odds of cardiovascular diseases, type 2 diabetes, metabolic syndrome, certain types of cancers, and becoming the victim of discriminatory behaviors.^1,2^ The economic burden of overweight and obesity includes rising health care costs, lost work, and premature deaths.^2^

Diets high in fruits and vegetables are protective against obesity^3^; conversely, unhealthful diets,^4^ including those with more sugar-sweetened beverages (SSBs) and added sugars from other sources,^5^ are positively associated with obesity. Studies have found African American families to purchase foods high in sugars, including SSBs in greater quantities than white households,^6^ and Latinos to report diets of higher quality compared with whites or African Americans.^7^ African Americans suffer from higher obesity rates than whites or Latinos.^8^

Dietary preferences and choices do not fully explain the burden of obesity on African American adults and children. One important factor put forth to explain these disparities is the “neighborhood context,”^9^ where, for example, the availability of fresh fruits and vegetables may be limited. In fact, the Healthy People 2030 framework recognizes the “neighborhood and built environment” as an important social determinant of health.^10^ Social determinants have been identified as the primary cause of heath inequities,^10^ and limited access to healthful food among certain racial/ethnic groups can perpetuate poorer diets, overweight and obesity, and the related negative health and quality of life outcomes.

The United States Department of Agriculture (USDA), Supplemental Nutrition Assistance Program Education (SNAP-Ed; “CalFresh Healthy Living” in California) provides individual and neighborhood-level interventions that include promoting the intake of fruits and vegetables and limiting the consumption of added sugars, as well as working with food retailers to offer more healthful food items, to individuals with household incomes ≤185% of the federal poverty level.^11^ The California Department of Social Services, which oversees the CalFresh Healthy Living program, supports the California Family Health Study (CFHS), an annual cross-sectional survey of mothers from CalFresh Healthy Living-eligible households across California. CFHS survey procedures include collecting detailed dietary information using a 24-h recall assessment. Supplemental survey items are used to record height and weight, and to ask mothers about the availability of healthful foods in their neighborhood.

The objectives of the current study were to investigate racial/ethnic inequities in dietary behaviors known to be associated with excess weight gain, overall diet quality, and obesity rates among white, Latina, African American, and Asian American, Native Hawaiian, or Pacific Islander (AANHPI) mothers from CalFresh Healthy Living-eligible households. We hypothesized that mothers from the racial/ethnic group demonstrating the poorest diet quality and highest rate for obesity would also report the most restricted access to fruits and vegetables and other healthful food in their neighborhood.

## Methods

Data for this study came from telephone interviews conducted in 2018 and 2019 with female adult caregivers of children 5–17 years from CalFresh Healthy Living-eligible households across California. Institutional Review Board approval was obtained from the California Health and Human Services Agency, Committee for the Protection of Human Subjects.

### Sampling and recruitment

The California Department of Health Services Medi-Cal (Medicaid in California) Eligibility Data System (MEDS) database was used to randomly select households with residents identified as speaking primarily English or Spanish from all 58 California counties. The MEDS includes individuals from households where ≥1 residents had applied for benefits administered by the State of California within the past year. Sampling procedures include removing from each frame those households with individuals who have participated in the CFHS during the previous year.

Using the names, mailing addresses, telephone numbers, and preferred language listed in the MEDS, selected households were sent a letter in English or Spanish that briefly described the study, followed by a telephone call by bilingual staff to confirm the youngest female caregiver of children, who we refer to here on as “mothers.” A $15 gift card was offered for survey participation. The data collection telephone interviews were scheduled with interested mothers at the end of each call.

Before the telephone interview, households were sent a pictorial food and beverage portion-size booklet, measuring cups and spoons, and a tape measure with instructions for assessing one's height. The cover letter included in the interview packets also asked mothers to record their weight and place both measures in a “location that will be easy to access when you are called for the interview.”

### Interviews

Trained interviewers conducted 24-h dietary recall interviews, in English or Spanish, using the National Cancer Institute's web-based Automated Self-administered 24-Hour (ASA24) Dietary Assessment Tool.^12^ The ASA24 involves asking respondents to identify all meals and snacks over the last 24 h, and then all foods and drinks consumed for each meal and snack. During this process, interviewers referenced the study-supplied portion-size booklet or measuring cups and spoons to guide mothers in identifying the quantity and size of each reported food and beverage item. All dietary intake information was entered into the web-based ASA24 system. Responses to standardized height and weight, access in their neighborhoods to fruits and vegetables and other healthy foods, and demographic questions were recorded into a computer-assisted telephone interviewing system.

### Outcome variables

Nine outcome variables were selected based on USDA SNAP-Ed Evaluation Framework^13^ indicators that are used by CalFresh Healthy Living to track levels of program-specific dietary behaviors, diet quality, and obesity rates, and identify determinants of these outcomes for program planning purposes, among the California SNAP-Ed eligible population.

Five ASA24-derived dietary outcome variables were examined in this study. *Cups of fruits and vegetables* represented intact whole or cut fruits and dark green, red, orange, starchy, and other vegetables, excluding legumes. *Ounces of SSBs* included sugar-sweetened soda, energy (e.g., Red Bull^®^), fruit (e.g., Sunny Delight^®^), sports (e.g., Gatorade^®^) drinks, and coffee or tea beverages. Excluded were beverages with artificial sweeteners or “diet” soda. *Teaspoons of added sugars* were white sugar, brown sugar, raw sugar, corn syrup, corn syrup solids, high fructose corn syrup, malt syrup, maple syrup, pancake syrup, fructose sweetener, liquid fructose, honey, molasses, dextrose, and dextrin, eaten separately or as ingredients in processed or prepared foods. *Kilocalories* from all foods and beverages consumed during the 24-h reporting period were calculated.

Healthy Eating Index (HEI)-2015 scores (theoretical range=0–100) were calculated based on established National Cancer Institute procedures^14^ from 13 components of dietary intake: total fruits; whole fruits; total vegetables; dark green vegetables and legumes; whole grains; total dairy; total protein foods; seafood and plant proteins; refined grains; added sugars; fatty acids; sodium; and total saturated fats. Higher scores equate to healthier diets, in accordance with the 2015–2020 Dietary Guidelines for Americans.^15^

Weight and height were assessed with the questions, “How much do you weigh without your shoes on?” and “How tall are you without your shoes on?” Body mass index (BMI) was calculated and mothers with a BMI of 30 or higher were considered obese. Rates for obesity excluded records based on available values for height and weight and criteria established by the Centers for Disease Control and Prevention.^16^

Mothers were asked to indicate their level of agreement (5-point scale: strongly agree to strongly disagree) to the statements, “In my neighborhood, it is easy to buy fresh fruits and vegetables” and “In my neighborhood, it is easy to buy healthy foods, such as low-fat milk and whole grain bread.” These items were acquired from Freedman and Bell,^17^ with the healthy foods item expanded to include the examples “such as low-fat milk and whole grain bread.” In their study, responses to the items were compared with the findings from audits of food stores within proximity of the survey participants. No significant differences were observed between participants' perceived access and the actual availability of fruits and vegetables or healthy foods, which the researchers interpreted as evidence of the validity of the measures.

### Demographics

Mothers' age was ascertained with the question, “What is your age?” Level of education was obtained by asking, “What is the highest level of school you have completed or the highest degree you have received?” Response options were, “8th grade or less,” “9th–12th grade (no diploma),” “high school graduate or GED completed,” “some vocational, trade, or business school but no diploma,” “completed a vocational, trade, or business school program,” “some college credit but no degree (including Associate's degree),” “college graduate 4 year degree,” or “postgraduate or professional degree.”

Mothers were coded as Latina if they responded “yes” to the question, “Are you of Hispanic, Latina, or Spanish origin?” The following questionnaire item was, “What is your race? You may answer more than one. Are you American Indian or Alaska Native, Asian, black or African American, Native Hawaiian or other Pacific Islander, white, or other?” Mothers identifying as African American were coded as such if they responded “no” to the Hispanic/Latina question; mothers were coded as white if not Hispanic/Latina or African American. Given their relatively small sample sizes, mothers with responses of AANHPI were combined into one racial/ethnic category.

### Analytic sample and statistical analyses

The ASA24 data and responses to the supplemental items were linked via unique study ID numbers. Over the 2-year study period, survey data were obtained from 9580 mothers. Excluded from all analyses were records from 66 mothers (0.69% of the sample) with reported race/ethnicity other than white, Latina, African American, or AANHPI, and 314 mothers (3.3%) with missing racial/ethnic data. The analyses of the 5 dietary behavior outcome variables omitted 354 mothers reporting implausible daily kilocalorie intake (≤400 or ≥4400 for adult women), per ASA-24 procedures.^12^ The BMI and obesity analyses were based on available height and/or weight values from 9034 mothers; these data were missing from 166 mothers, or 1.8% of the analytic sample. Latinas (1.2%) were most likely to have missing height and/or weight data, followed by African Americans (1.2%), whites (1.1%), and AANHPIs (0.0%).

Mean values for intake of cups of total fruits and vegetables; ounces of SSBs; teaspoons of added sugars; kilocalories; and HEI-2015 scores were compared for white, Latina, African American, and AANHPI mothers using general linear models (ANOVA) with Fisher's least significant difference tests for *post hoc* mean comparisons. White mothers served as the reference group for linear regression analyses for BMI and logistic regression analyses examining obesity and limited perceived access to fresh fruits and vegetables and healthy foods in one's neighborhood (responses of “disagree” and “strongly disagree” coded as 1; 0 otherwise). All models adjusted for the covariates of age (centered on the mean) and education (less than a high school education as the reference group to high school graduate or vocational school=1; 0 otherwise; or graduate schooling=1; 0 otherwise). Data merging, cleaning, coding, and analyses were conducted with SPSS (version 25.0; IBM Corp, Armonk, NY).

## Results

The analytic sample of 9200 mothers was 66.3% Latina, 17.3% white, 12.6% African American, and 3.8% AANHPIs. The average age of mothers was 38.3 years (median=38.0 years). Less than a high school education was reported by 29.2% of mothers, 31.7% had completed high school, and 39.1% had graduate schooling.

Differences were observed across the four racial/ethnic groups for cups of fruits and vegetables, ounces of SSBs, teaspoons of added sugars, daily kilocalorie intake, and HEI-2015 scores (*p*<0.001 for all *F* statistics). As seen in Table 1, Latinas and AANHPIs ate greater quantities of fruits and vegetables than whites (*p*s<0.01); African Americans reported the lowest level for intake of fruits and vegetables (*p*s<0.05).

**Table 1.** Adjusted general linear models for mother's dietary behaviors and diet quality by race/ethnicity, California Family Health Study, 2018–2019

	White (*n*=1524), adjusted mean (95% CI)	Latina (*n*=5890), adjusted mean (95% CI)	African American (*n*=1093), adjusted mean (95% CI)	Asian American, Native Hawaiian, or Pacific Islander (*n*=337), adjusted mean (95% CI)
Fruits and vegetables, cups	2.87^b^ (2.75–2.99)	3.13^c^ (3.06–3.19)	2.68^a^ (2.54–2.82)	3.25^c^ (3.00–2.51)
Sugar-sweetened beverages, ounces	8.5^b,c^ (7.8–9.1)	7.8^b^ (7.5–8.2)	9.1^c^ (8.4–9.9)	5.6^a^ (4.3–6.9)
Added sugars, teaspoons	12.8^c^ (12.2–13.5)	11.2^b^ (10.9–11.6)	13.7^d^ (13.4–14.8)	9.5^a^ (8.3–10.7)
Kilocalories	1827^a^ (1789–1865)	1789^a^ (1770–1808)	1896^b^ (1852–1940)	1826^a,b^ (1748–1904)
Healthy eating index-2015 scores^*^	54.1^a^ (53.4–54.8)	58.6^c^ (58.2–59.0)	53.8^a^ (53.0–54.7)	56.6^b^ (55.1–58.0)

Covariates were age and level of education. Means with differing superscripts are significantly different at *p*<0.05.

^*^ Higher values equate to healthier diets.

CI, confidence interval.

African American mothers' daily dietary intake included more ounces of SSBs than Latinas and AANHPIs (*p*s<0.01), and the highest quantity of added sugars than mothers from the other three racial/ethnic groups (*p*s<0.05). Daily kilocalorie intake was greater for African Americans than whites and Latinas (*p*s<0.05). Finally, poorer levels of diet quality, as signified by lower HEI-2015 scores, were found for African American and white mothers (*p*s<0.01).

African American mothers had a higher unadjusted and adjusted mean BMI (both *p*s<0.001) than white, Latina, and AANHPI mothers (Table 2). The overall unadjusted obesity rate for CalFresh Healthy Living-eligible mothers across California was 45.7%. The unadjusted rate of obesity among African American mothers was 54.7%, compared with 39.9% for white mothers, 46.9% for Latina mothers, and 23.5% for AANHPI mothers. The adjusted odds of obesity for Latina compared with white mothers was 1.25 (95% confidence interval [CI]=1.11–1.41), and for African American mothers was 1.82 (95% CI=1.56–2.12) over white mothers. AANHPI mothers were half as likely as white mothers to be obese (adjusted odd ratio [aOR]=0.46; 95% CI=0.36–0.61).

**Table 2.** Adjusted regression models for mother's body mass index, obesity status, and perceived access to fruits and vegetables and healthy foods by race/ethnicity, California Family Health Study, 2018–2019

	Body mass index, *n*=9034		Obese, *n*=9034		Limited perceived access to fresh fruits and vegetables in one's neighborhood, *n*=9200		Limited perceived access to healthy foods in one's neighborhood, *n*=9200	
	Unadjusted mean	Adjusted mean (95% CI)	Unadjusted, %	Adjusted OR (95% CI)	Unadjusted, %	Adjusted OR (95% CI)	Unadjusted, %	Adjusted OR (95% CI)
White	29.6	29.9 (29.4–30.4)	39.9	Reference	9.4	Reference	6.1	Reference
Latina	30.6	30.7 (30.3–31.1)	46.9	1.25 (1.11–1.41)	9.5	1.02 (0.83–1.24)	5.6	0.91 (0.71–1.16)
African American	32.2	32.5 (32.0–33.1)	54.7	1.82 (1.56–2.12)	16.1	1.87 (1.48–2.35)	11.9	2.10 (1.60–2.76)
Asian American, Native Hawaiian, or Pacific Islander	26.3	26.6 (25.8–27.5)	23.5	0.46 (0.36–0.61)	6.0	0.61 (0.38–0.99)	3.2	0.50 (0.27–0.94)

Covariates were age and level of education.

OR, odds ratio; CI, confidence interval.

Limited perceived availability of fresh fruits and vegetables was reported for a greater proportion of African American mothers (aOR=1.87; 95% CI=1.48–2.35). Limited perceived availability to healthy foods in general was twice as likely to be reported by African American mothers (aOR=2.10; 95% CI=1.60–2.76).

In subsequent analyses, we examined the relationships between responses to the two food access items and obesity. Regardless of race/ethnicity, age, or level of education, mothers who reported limited perceived availability of fruits and vegetables (aOR=1.28; 95% CI=1.11–1.47) or healthy foods in general (aOR=1.40; 95% CI=1.18–1.66) were more likely to be obese.

## Discussion

We found African American mothers to consume more ounces of SSBs per day and report poorer diet quality than Latina and AANHPI mothers. Total kilocalories per day were also higher for African Americans compared with white and Latina mothers. Moreover, white, Latina, and AANHPI mothers' daily intake of fruits and vegetables was greater, and teaspoons of added sugars were less, compared with African American mothers.

We compared these findings with similar dietary components of the HEI-2015 as assessed from a representative sample of U.S. adults. Results from the 2017 to 2018 National Health and Nutrition Examination Survey (NHANES)^18^ indicate that African American adults consume lower quantities of fruits than AANHPI adults, higher levels of added sugars than Latino and AANHPI adults, and fewer vegetables than white, Latino, and AANHPI adults. Data from our population-based sample of mothers from low-income California households found unhealthier levels for intake of fruits and vegetables and added sugars by African Americans compared with mothers from all other racial/ethnic groups.

While levels of overall diet quality among female NHANES^18^ participants identifying as white, Latino, or African American were statistically similar, African American mothers participating in the 2018–2019 CFHS surveys had lower HEI-2015 scores than Latina and AANHPI mothers. In short, among mothers from low-income households across California, we found inequities for African Americans as related to those healthful and unhealthful dietary behaviors recognized by the USDA^13^ as key indicators for obesity prevention among the SNAP-Ed eligible population.

Accordingly, our analysis revealed that over half of African American mothers from CalFresh Healthy Living-eligible households were obese, with a rate higher than for white, Latina, and AANHPI mothers, ever after adjusting for mothers' age and level of education. A recent systematic review of studies reporting obesity rates in the United States also found a higher proportion of African Americans to be obese than women from other racial/ethnic groups.^19^

The hypothesis for the current study was supported. A higher proportion of African American mothers, compared with white, Latina, and AANHPI mothers, reported restricted perceived access to fruits and vegetables and healthful food in general in their neighborhoods. In fact, African Americans were over twice as likely to disagree with the statement that it was easy to buy healthy foods in their neighborhood compared with whites and Latinas.

It would be naive to suggest that increasing the availability of outlets that offer healthful food and beverage items would lead to improved dietary behaviors and reduce obesity rates within African American communities. In fact, two studies examining the effects of new grocery stores in low-income neighborhoods versus comparison communities reported relevant findings. In one study, perceptions of food accessibility did increase in the intervention neighborhood, but intake of fruits and vegetables and BMI remained unchanged among the predominantly African American adult residents over the study period.^20^ In the second study, no differences were observed for the availability of healthful foods at home or children's dietary intake.^21^

In a critical assessment of five intensive, longitudinal, state-of-the-art obesity-prevention studies with findings of no differences in body mass for interventions compared with controls, Dietz identified a host of adverse community conditions other than limited access to healthful food, that likely restricted the potential impact on weight gain among the selected high-risk, low-income populations.^22^ Scholars are increasingly pointing out that these types of conditions are a result of and continue to be perpetuated by systemic racism,^23–25^ which encompasses, as initially conceptualized by Feagin,^26^ three factors: structural (whites controlling government and commercial institutions); disproportionate distribution of resources; and social psychological advantages (the belief in white superiority).^21^

Neighborhood disadvantage often results from limited income, poverty, and unemployment, that is, socioeconomic factors.^23,24^ Phelan and Link argue that socioeconomic status is a fundamental cause of health disparities, and differences in levels of socioeconomic status across racial/ethnic groups are caused by racism.^24^ Furthermore, they suggest that racism is related to health outcomes independent of socioeconomic status, due to “flexible” or “health-enhancing” resources (e.g., money, knowledge, power, social connections). In turn, the availability of health-enhancing resources, those associated with the three factors of systemic racism, influence multiple mechanisms, including neighborhood conditions such as limited access to healthful foods.

In summary, one's neighborhood can influence dietary behaviors and health outcomes by lack of availability of healthful foods and socioeconomic barriers for travel to and purchase healthful items at grocery stores, as examples. Addressing these two factors alone within African American communities is extremely challenging. Yet, the consequence of unhealthful dietary behaviors and their outcomes such as obesity, as examined in the current study, and others such as cardiovascular disease, will not be sufficiently addressed until the factors responsible for systemic racism are adequately addressed.

Strengths of the current study were population-based sampling and administration of a validated 24-h dietary recall assessment to calculate precise measures of components of low-income mothers' daily food and beverage intake and overall diet quality. Rather than use zip codes or other predefined geographic areas to approximate mothers' neighborhood, our methodology allowed for responses to the perceived access items to be based on one's individual assessments of what constitutes her residential environment. A perceived rather than inferred neighborhood context also represents a study strength.

Limitations include biases known to occur when behaviors and anthropometric data are self-reported, the assumption that dietary intake during the study recall period of the previous day represents mothers' typical daily consumption behaviors, and the absence of other environmental factors other than availability of healthful food that could encourage unhealthful dietary behaviors, such as the availability of fast food restaurants and marketing efforts to promote the consumption of SSBs.

In addition, the two perceived access variables have undergone limited validity tests. The CFHS is designed specifically to track USDA SNAP-Ed population-level evaluation framework indicators,^24^ with a survey instrument purposely including limited demographic items and excluding questions related to items such as income and receipt of publicly funded benefits. These measures would have been beneficial as covariate to control for potential confounding between the associations of race/ethnicity and the outcomes examined in this study.

Finally, our agreement with the California Department of Social Services, which supports the CFHS through USDA funds, is that the analytic team can only use addresses to remove survey respondents who were interviewed in the previous year. Without this restriction, the addresses of the mothers who participated in the 2018 and 2019 CFHS could have been used to create one or more geographic location variables as a proxy to control for mothers' residences in relationship to available grocery outlets.

## Conclusion

Accessible neighborhood stores that offer healthful food and beverage items within African American communities represent a necessary but not sufficient means of improving dietary behaviors and addressing elevated body mass. Strategies and resources beyond that are commonly conceived by public health nutrition practitioners and policy makers, including those affiliated with SNAP-Ed, must be incorporated into interventions aimed at African American families. These broader interventions must address socioeconomic barriers to health equality, such as providing low-income families with a living wage, and change the beliefs and behaviors that continued to foster systemic racism.

## Abbreviations Used

AANHPI: Asian American, Native Hawaiian, or Pacific Islander
ANOVA: analysis of variance
aOR: adjusted odds ratio
ASA24: Automated Self-administered 24-Hour
BMI: body mass index
CFHS: California Family Health Study
CI: confidence interval
HEI: Healthy Eating Index
MEDS: Medi-Cal (Medicaid in California) Eligibility Data System
NHANES: National Health and Nutrition Survey
OR: odds ratio
SNAP-Ed: Supplemental Nutrition Assistance Program Education
SSB: sugar-sweetened beverage
USDA: United States Department of Agriculture
